# Supplementary material for: Drug–Drug Interactions and Pharmacogenomic Evaluation in Colorectal Cancer Patients: The New Drug-PIN® System Comprehensive Approach
Source: Pharmaceuticals (Basel). 2021 Jan 15;14(1):67. doi: 10.3390/ph14010067 (PMC7830292; doi:10.3390/ph14010067)
Supplement: Supplementary file 1 [file pharmaceuticals-14-00067-s001.pdf]

pharmaceuticals

Table 1. supplementary. Panel of SNPs investigated.

Gene	Rs Code	Polymorphism	Amino acid	Genotype	Functional effects
**Transporters**					
**ABCB1/MDR1**	rs1128503<br>rs1045642	C1236T<br>C3435T	Gly412Gly<br>Ile1815=	**T allele leads to lower activity**	Decreased
**ABBC1/MRP1**	rs45511401	G671V-G>T	Gly671Val		
**ABCB1**	rs4148738	G>A	Intron	A allele leads to increased Dabigatran concentration	
**ABCC2/MRP2**	rs8187710<br>rs17222723	G4544A<br>T3563A	Cys1515Tyr<br>Val1188Glu	A allele leads to lower activity	Decreased
**ABCC2 24C>T**	rs717620	C5116T	Promoter	T allele leads to lower expression	Lower expression
**ABCG2**	rs2231142	C421A	Gln141Lys	**A** allele leads to lower expression	Lower expression
**SLC15A2-L350F**	rs2257212	C>T	Leu350Phe	**-**	-
**SLCO1B1**	rs4363657	T521C	Intron	**C** allele leads to lower activity	Decreased
	rs4149056	T>C	Val174Ala		
**5HTT-LPR**		Repeat region	Promoter	S allele leads to lower transcription	
**CYP**					
**CYP1A2*1C**	rs2069514	G3860A	None	A allele leads to lower activity	Decreased
**CYP1A2*1F**	rs762551	C163A	Intron	A allele leads to increased inducibility	Increased
**CYP2C19*17**	rs12248560	C806T	Upstream	Heterozygous	Increased
**CYP2C19*10**<br>**CYP2C19*2**<br>**CYP2C19*3**<br>**CYP2C19*4**	rs6413438<br>rs4244285<br>rs4986893<br>rs28399504	C680T<br>G681A<br>G636A<br>A5026G	Pro227Leu<br>Pro227=<br>Trp212Ter<br>Met1Val	T/A/G alleles leads to lower activity	Deceased
**CYP2C19*5**<br>**CYP2C19*7**	rs56337013<br>rs72558186	C1297T<br>T24319A	Arg433Trp	Homozygous	Increased

			Splice Donor		
**CYP2C9*2**<br>**CYP2C9*3**	rs1799853<br>rs1057910	C430T<br>A1075C	Cys144Arg<br>Ile359Leu	T/C allele leads to lower activity	Decreased
**CYP2D6*2**<br>**CYP2D6*XN**	rs16947<br>-	C2850T<br>-	Arg296Cys<br>Amplificatio n	Homozygous<br>-	Increased
**CYP2D6*10**<br>**CYP2D6*17**<br>**CYP2D6*6**<br>**CYP2D6*20**	rs1065852<br>rs28371706<br>rs5030655<br>rs72549354	C100T<br>C1023T<br>1707delT<br>insG	Pro34Ser<br>Thr107Ile<br>Trp152fs<br>Leu213fs	**Heterozygou s**	**Decreased**
**CYP2D6*5**<br>**CYP2D6*38**	-<br>rs72549351	Deletion<br>delGACT	Stop codon<br>Thr272fs	-	Decreased
**CYP2D6*29**	rs61736512	G1659A	Val136Met	A allele leads to lower activity	Decreased
**CYP2D6*2A**<br>**CYP2D6*3**<br>**CYP2D6*4**<br>**CYP2D6*41**<br>**CYP2D6*7**<br>**CYP2D6*9**	rs1080985<br>rs35742686<br>rs3892097<br>rs28371725<br>rs5030867<br>rs5030656	C1584G<br>2549delA<br>G1846A<br>G2988A<br>A2935C<br>2615-2617delAAG	Upstream<br>Arg259fs<br>Acceptor<br>Intron<br>His324Pro<br>Inframe del.	Homozygous	Decreased
**CYP3A4*1B**	rs2740574	A392G	Upstream	Homozygous	Decreased
**CYP3A4*22**	rs35599367	C15389T	Intron	G alleles lead to increased activity	Increased
**CYP3A5*3**	rs776746	A6986AG	Acceptor	Heterozygou s	Decreased
**CYP1A1**	rs1048943	T>C	Ile462Val	C allele leads to increased activity	Increased
**CYP2B6**	rs2279343<br>rs3745274<br>rs3211371<br>rs28399499	A785G<br>G516T<br>C1459T<br>T 983C	Lys262Arg<br>Gln172His<br>Arg487Cys<br>Ile328Thr	G/T allele leads to lower activity	Decreased
**CYP2A6**	rs28399433<br>rs1801272	A48C<br>T>A		C/A allele leads to	Decreased

				lower expression	
**CYP2C8*2**	rs11572103	A805T	Ile269Phe	-	-
**CYP2C8*4**	rs1058930	C792G	Ile264Met	C allele leads to lower activity	Decreased
**Other drug metabolizing enzymes**					
**TPMT*2**	rs1800462	C>G	Ala80Pro	C allele leads to lower activity	Decreased
**TPMT*3B**<br>**TPMT*3C**	rs1800460<br>rs1142345	G460A<br>A719G	Ala154Thr<br>Tyr240Cys	Heterozygou s	Decreased/Inactive
**UGT1A1*28**	rs8175347	(TA)(n) repeat sequence	TATA box	6/7 repeat	Decreased
**UGT2B17**		Delection		Heterozygou s	Decreased
**DPYD**	rs3918290	IVS14+1G>A	Splice donor	Heterozygou s	Decreased
	rs67376798	A2846T	Asp949Val	Homozygous	
**DPYD*6**	rs1801160	G>A	Val732Ile	A allele leads to lower activity	Decrease
**DPYD*9A**	rs1801265	T>C	Cys29Arg	-	-
**DPY**	rs2297595	A166G	Initiator Codon	-	-
**COMT**	rs4680<br>rs4633	G>A<br>C>T	Val158Met<br>His62His	A/T alleles lead to lower activity	Decreased
**COMT**	rs4818	C>G	Leu136Leu	G alleles lead to increased activity	Increased
**MTHFR**	rs1801133<br>rs1801131	C677T<br>A1298C	Ala222Val<br>Glu429Ala	**T/C alleles lead to lower activity**	Decreased
**TYMS**	rs45445694	TSER*2/TSER* 3	28 bp repeat in enhancer region	Increased transcription	Increased
	rs2853542<br>rs34743033	TSER*3R G/C	second repeat of 3R allele	Reduced transcription	Decreased

Abbreviation: ABCB1, ATP binding cassette subfamily B member 1; ABCC1, ATP Binding Cassette Subfamily C Member 1; MRP1, Multidrug resistance-associated protein 1; ABCC2, ATP Binding Cassette

	rs151264360<br>rs869066439	1494del6b	I/D of TTAAAG sequence on the 3'- UTR	TYMS mRNA instability	Decreased
**NAT1*14B**	rs4986782	G560A	Arg187Gln	A allele leads to lower activity	Decreased
**NAT1*15B**	rs5030839	C599T	Stop Codon	-	Inactive
**NAT1*17B**	rs56379106	C190T	Arg64Trp	C alleles lead to lower activity	Decreased
**NAT1*22B**	rs56172717	A752T	Asp251Val	T alleles lead to lower activity	Decreased
**NAT2*3**<br>**NAT2*6**<br>**NAT2*7**	rs1801280<br>rs1799930<br>rs1799931	T341C<br>G590A<br>G857A	Ile114Thr<br>Arg197Gln<br>Gly286Glu	C/A alleles lead to lower activity	Decreased
**UMPS**	rs1801019	G213A<br>G>C	Gly213Asp<br>Gly213Ala	-	-
**CBS**	rs234706	C699T	-	Allele T associated with increased risk of ammonia and sulphite detoxification defects	
**Receptors**					
**DRD2/ANKK1-Q713K**	rs1800497	C957T		Heterozygous	Reduced dopamine binding sites
**DRD2-141-insC/delC**	rs1799732	insC/delC	Upstream	Heterozygous	Reduced receptor expression
**DRD2-S311C**	rs1801028	C932G	Ser311Cys	Heterozygous	Decreased affinity for dopamine
**DRD3-S9G**	rs6280	A25G	Gly9Ser	Homozygous	Normal
**HTR2A**	rs6314<br>rs7997012<br>rs6311	C1354T<br>A>G<br>G1438A	His452Ty<br>Intron<br>Upstream	Heterozygous<br>Homozygous<br>Homozygous	-<br>-<br>-

Subfamily C Member 2; MRP2, Multidrug resistance-associated protein 2; ABCG2, ATP-binding cassette subfamily G membro 2; SLC15A2, Solute Carrier Family 15 Member 2; SLCO1B1, solute carrier organic anion

**HTR2C**	rs6318	G68C	Cys23Ser	Homozygous	Increased cardiovascular events
**OPRM1**	rs1799971	A118G	Asn40Asp	Heterozygous	More pain
**Oxidation reduction enzymes**					
**GSTP1**	rs1695	A313G	Ile105Val	C allele leads to lower activity	Decreased
**GSTM1**<br>**GSTT1**		Deletion		Heterozygous	Inactive
**DNA repair enzymes**					
**ERCC1**	rs3212986<br>rs11615	C8092A<br>T19007C	3 Prime UTR<br>Asn118=	-	-
**XRCC1**	rs25487	G28152A	Gln399Arg	Heterozygous	Decreased
**Lipid metabolism**					
**APOA1**	rs1799837	G75A		Protective against cardiovascular event	
**APOB1**	rs5742904	G10580A	Arg3500Gln	Heterozygous	Hypercholesterolemia
**CETP**	rs1532624	C>A	Intron	-	-
**HMGCR**	rs3761740<br>rs5908	C911A<br>A>G	Upstream tra.<br>Ile585Val	-	-
**PON1**	rs662<br>rs705379	A575G<br>C108T	Gln192Arg<br>Upstream	G/T alleles lead to lower activity	Decreased
**Coagulation enzymes**					
**VKORC**	rs9923231<br>rs9934438	G1639A<br>C1173T	Upstream<br>Intron	A/T alleles lead to less coagulating capacity	Decreased
**CES1**	rs2244613<br>rs8192935	A>C<br>C>T	Intron	C allele leads to decreased Dabigatran concentration	
**F II**	rs1799963	G20210A	3 Prime UTR	-	-
**PAI**	rs1799889	4G/5G	Upstream	Heterozygous	Increased risk of thrombotic events

**F V**	rs6025	G1691A	Arg506Gln	A allele Increased risk of thrombotic events	
**β-fibrinogen**	rs1800790	G455A	-	-	-
**FattoreXIII**	rs5985	G>T	Val135Leu	-	-

transporter family member 1B1; 5-HTTLPR, serotonin-transporter-linked polymorphic region; CYP1A2, Cytochrome P450 Family 1 Subfamily A Member 2; CYP2C19, cytochrome P450 family 2 subfamily C member 19; CYP2D6 Cytochrome P450 family 2 subfamily D member 6; CYP3A4, Cytochrome P450 family 3 subfamily A member 4; CYP3A5, Cytochrome P450 family 3 subfamily A member 5; CYP1A1 Cytochrome P450 family 1 subfamily A member 1; CYP2B6 Cytochrome P450 family 2 subfamily B member 6; CYP2C8, Cytochrome P450 family 2 subfamily C member 8; TPMT, Thiopurine methyltransferase; UGT1A1, uridine-diphosphoglucuronate glucuronosyltransferase; DPYD, dihydropyrmidinase; COMT, catechol O-methyltransferase; TSER, synthase enhancer region; MTHFR, methylenetetrahydrofolate reductase; TYMS, thymidylate synthase; NAT1, Arylamine N-acetyltransferase 1; NAT2, Arylamine N-acetyltransferase 2; UMPS, Uridine Monophosphate Synthetase; DRD2, dopamine receptor D2; ANKK1, Ankyrin repeat and kinase domain containing 1; HTR2A, 5-Hydroxytryptamine Receptor 2A; HTR2C, 5-Hydroxytryptamine Receptor 2C; OPRM1, Opioid Receptor Mu 1; GSTP1, Glutathione S-Transferase P1; GSTM1, glutathione S-transferase Mu 1; GSTT1, Glutathione S-Transferase Theta 1; ERCC1,Excision Repair 1, Endonuclease Non-Catalytic Subunit; XRCC1, X-ray repair cross complementing protein 1; APOA1, Apolipoprotein A1; APOB1, Apolipoprotein B1; CETP, Cholesteryl ester transfer protein; HMGCR, 3-Hydroxy-3-Methylglutaryl-CoA Reductase; PON1, paraoxonase/arylesterase 1; VKORC, Vitamin K epOxide Reductase Complex; CES1 Carboxylic ester hydrolase; F II, coagulation factor II; PAI, Plasminogen Activator Inhibitor; F V, coagulation factor V; CBS, cystathionine beta-synthase; F XIII, coagulation factor XIII;
